# Barriers and facilitators to community-based, peer-led sexual and reproductive health intervention for adolescent girls and young women in northeastern Uganda: A qualitative study

**DOI:** 10.1371/journal.pgph.0005828

**Published:** 2026-06-11

**Authors:** Alimah Komuhangi, Rogers Kajabwangu, Jonathan Izudi

**Affiliations:** 1 Afya na Haki Institute, Faculty of Health Sciences, Canaan Sites, Gayaza, Uganda; 2 School of Public Health, Clarke International University, Kampala, Uganda; 3 Texila American University, Georgetown, Guyana, South America; 4 Department of Obstetrics and Gynecology, Mbarara Regional Referral Hospital, Mbarara, Uganda; 5 Division of Urogynecology and Female Pelvic Floor Medicine, Mbarara University of Science and Technology, Mbarara, Uganda; 6 Directorate of Graduate Training, Research and Innovations, Muni University, Arua, Uganda; 7 Department of Community Health, Faculty of Medicine, Mbarara University of Science and Technology, Mbarara, Uganda; London School of Hygiene and Tropical Medicine, UNITED KINGDOM OF GREAT BRITAIN AND NORTHERN IRELAND

## Abstract

Peer-led interventions can effectively improve sexual and reproductive health (SRH) knowledge, promote safer sexual behaviors among adolescents and young people, especially where health system capacity is limited. To inform a planned pre-post study, we explored the barriers and facilitators to the implementation of a community-based, peer-led SRH intervention for adolescent girls and young women (AGYW) aged 15–24 years in northeastern Uganda. We designed a qualitative case study and collected data through focus group discussions across AGYW in six categories: in-school, out-of-school, ever given birth, and never given birth, aged 15–17 years, and aged ≥18 years. We also conducted key informant interviews with SRH officials in the district and at two health facilities. Data collection was guided by the socioecological model. We performed thematic analysis and reported findings using themes along with the participants’ quotes mapped onto the socioecological model. A total of 51 adolescent girls and young women, and five key informant interviews with the district and health facility level officials were conducted. Findings show that the anticipated barriers and facilitators to implementation occur across socioecological levels. At the individual level, stigma and fear of judgment hindered participation, whereas trust in peers supported engagement. Limited autonomy hindered access at the interpersonal level, but support from family and friends helped. Institutional barriers included judgmental provider attitudes and confidentiality concerns; existing youth-friendly services and school clubs were facilitators. At the community level, myths, misconceptions, and restrictive cultural norms posed challenges, while community dialogues and champions encouraged acceptance. No major policy barriers were noted; however, supportive SRH policies and existing partnerships were facilitators. The implementation of the intervention may be influenced by several factors across individual, interpersonal, institutional, community, and policy levels. Future implementation should consider addressing the barriers while leveraging the facilitators to enhance implementation outcomes among AGYW.

## Introduction

Adolescent sexual and reproductive health (SRH) remains a pressing public health concern in low- and middle-income countries, where adolescent girls and young women (AGYW) face disproportionate risks and unmet SRH needs. Poor SRH outcomes among AGYW contribute to school dropout, early marriage, and intergenerational cycles of poverty. In Uganda, for instance, 25% of adolescent girls aged 15–19 have begun childbearing, and over 13% are infected with at least one sexually transmitted infection (STI), including human immunodeficiency virus (HIV) [1]. These challenges are particularly acute in rural and marginalized regions such as Moroto District in the Karamoja sub-region of northeastern Uganda.

Uganda’s policy framework, such as the National Adolescent Health Policy (2016/2021), the National Adolescent Health Strategy (2018–2025), and the integration of Adolescent and Youth-Friendly Services into national health packages, underscores the government’s commitment to adolescent SRH. However, despite these policies, AGYW in northeastern Uganda continue to face barriers including limited access to accurate SRH information, stigma, and constrained service availability. These challenges are compounded by the weak health system in the region [2], a prolonged (over four decades) history of insecurity, nomadic pastoralists’ lifestyle, and cultural barriers to SRH services uptake [3].

Evidence suggests that peer-led interventions can effectively improve SRH knowledge, promote safer sexual behaviors, and enhance service uptake among adolescents, especially where health system capacity is limited [4], and cultural sensitivities constrain open discussions about sexuality. We propose to implement a community-based peer-led sexual and reproductive health education package to reduce risky sexual behavior and improve SRH knowledge among AGYW in northeastern Uganda. However, the conditions (implementation determinants) under which the peer-led interventions can be effectively implemented remain poorly understood.

To address this gap, we designed a qualitative study to explore the barriers and facilitators to implementing a peer-led SRH educational intervention to improve comprehensive SRH knowledge and reduce risky sexual behaviors among AGYW in northeastern Uganda. The intervention is designed as a series of four structured modules to be delivered through participatory sessions conducted in both English and the local language *(Ngakarimojong)* to ensure cultural and linguistic accessibility. The modules are intended to address the prevention of risky sexual behaviour; prevention of unintended pregnancies, unsafe abortions, and HIV/STI transmission; gender-based violence and communication skills; and contraceptive methods alongside sexual-health counselling.

The sessions are planned to combine biomedical information with culturally contextualised discussions, role-plays, storytelling, and peer dialogues based on locally relevant scenarios such as early marriage and transactional sex. In addition, the intervention is designed to incorporate life-skills training to strengthen safer sexual practices (including condom use), refuse unwanted or coerced sexual advances, and make informed decisions regarding their sexual and reproductive health, such as contraceptive use and seeking appropriate health services, while providing demonstrations of modern contraceptive options and information on referral pathways to youth-friendly health services.

All sessions are intended to be delivered by trained female peer educators from the same communities as participants, using participatory learning approaches. Findings from this formative work will inform the design of a pre-post quasi-experimental study with a nonequivalent comparison group to evaluate the effectiveness of the intervention in the real world.

## Methods and materials

### Study design and setting

This qualitative case study was conducted across seven villages in Moroto district in northeastern Uganda. The district is predominantly rural and comprises pastoralists with an estimated population of 103,639 based on the most recent national survey estimates [1]. The seven villages included Bazar, Camp Swahili, Kakoliye, Labour Line, Nakapelimen, Natumukasko, and Singila. These villages were purposively selected based on several considerations: (1) the presence of a high concentration of adolescent girls and young women (AGYW), (2) documented vulnerabilities related to early marriage, school dropout, and limited access to sexual and reproductive health (SRH) services, and (3) the selected villages also represent both peri-urban and rural settlements within Moroto Municipality, allowing the study to capture diverse contextual experiences of AGYW within the district. In addition, Adolescent fertility in this setting remains high at 23.6%, and modern contraceptive use is extremely low at 7.3% [5]. The district is among the least socio-economically developed in the country, with an estimated literacy rate of about 55%, which is 12 percentage points lower than the national literacy rate average of 67%. About three in every 10 adolescents in the district drop out of school compared with the national average of two in every 10 adolescents [6].

The setting has strong male-controlled cultural norms that value early marriage and high fertility as a source of wealth and social prestige [7]. The commonest vulnerabilities among AGYW include early marriages, transactional sex, low educational attainment, limited autonomy concerning SRH issues, and high rates of alcohol and substance use. These challenges occur within a context shaped by strong male-dominated traditions, geographic isolation, and under-resourced health systems [8].

### Study population and sampling

Two categories of participants were included in the study: AGYW and district and health facility-level officials as key SRH stakeholders. Eligible AGYW were aged 15–24 years, had lived in the district for at least six months to ensure familiarity with the local social and health context. They were disaggregated into four distinct groups to obtain diverse views and perspectives. The groups included in-school, out-of-school, ever given birth, and never given birth, aged 15–17 years, and aged ≥18 years. AGYW aged below 18 years and had no parent or guardian to provide assent, or who had a medical documentation of a mental health problem, were excluded from the study. District and health facility participants included those actively engaged in the SRH programming and provision, and they included the District Health Officer, the Assistant District Health Officer in charge of maternal and child health, the District Community Development Officer, and two healthcare providers, including the focal person for SRH across two health facilities.

These individuals oversee district-level SRH programming and have valuable perspectives on contextual factors that may influence the delivery of SRH interventions, hence their inclusion in the study. We employed a purposive sampling approach to select the AGYW and the district and health facility officials to gain in-depth and context-specific insights into SRH.

### Sample size determination

The sample size was guided by the principle of thematic saturation [9], which was reached when additional interviews yielded no new themes or insights across the participants. Although thematic saturation guided the study, a *priori* sample size based on methodological recommendations for achieving the saturation was 4–7 focus group discussions (FGDs) with AGYW and 3–6 key informant interviews (KIIs) with key stakeholders.

### Data collection approach

Data (S1 Data) were collected between 13 September 2024 and 11 October 2024 through six focus group discussions (FGDs) with AGYW and five face-to-face key informant interviews (KIIs) with the district and health facility level officials. Prior to the data collection, the FGDs and KII guides were pretested among eight AGYW and two health workers in Rupa subcounty. This allowed the research team to assess clarity, flow, cultural appropriateness, and comprehension of the questions. Feedback from the pretest informed refinement of question wording, sequencing, and probing strategies to enhance the quality and depth of data obtained. For the data collection, the participants were purposively stratified based on characteristics likely to influence SRH experiences, including age (15–17 years vs. ≥ 18 years), schooling status (in-school vs. out-of-school), and parity (ever given birth vs. never given birth). Following recruitment, participants were allocated into FGDs to ensure homogeneity within groups, with each group comprising individuals sharing similar attributes across these stratification variables. For instance, separate FGDs were conducted for in-school adolescents aged 15–17 years who had never given birth, and for out-of-school young women aged ≥18 years who had ever given birth. This approach minimized power imbalances and enabled participants to freely discuss sensitive SRH issues within peer groups of similar maturity and lived experiences. All participants were informed of the study purpose, the voluntary nature of participation, and their right to withdraw at any time without consequences.

Data were collected for 45 minutes to an hour by two trained research assistants, a male Public Health Specialist and a female Social Scientist, each with 5–7 years of experience in qualitative research. All key informant interviews (KIIs) were conducted in English within the offices of district and health facility officials to ensure privacy and confidentiality. In contrast, FGDswere conducted in the local language *(Ngakarimojong)* at mutually agreed community venues such as schools, churches, and open spaces. All interviews and discussions were audio-recorded using a portable digital recorder, and detailed field notes were taken by the research assistants and summarized within 24 hours with respective codes. We did multiple close readings of the transcripts, allowing us to become fully familiar with the content and gain a comprehensive appreciation of the participants’ perspectives. An initial inductive coding process was undertaken by the two research assistants, allowing codes to emerge directly from the data rather than being predetermined.

### FGD and KII topic guides informed by the socioecological model

We conducted KIIs and FGDs and the topics were guided by the socioecological model (SEM) domains [10]. The SEM was used to guide data collection and analysis, as barriers and facilitators to implementing peer-led SRH interventions among AGYW operate across multiple levels, including individual, interpersonal, community, structural, and policy contexts,ive domains. While the five domains are analytically distinct, they are empirically interconnected. At the individual-level, the topics discussed focused on personal agency, perceived value of SRH education, and trust in peers. The topics at the interpersonal level included the influence of family members, peers, and intimate partners, and the topics at the institutional level included access to and experience with youth-friendly services, school-based support, and Non-Governmental Organisation (NGO) involvement. At the community level, the topics included existing cultural beliefs, social norms, and stigma about SRH, while policy level topics encompassed the availability and implementation of national and district-level adolescent SRH policies. Additional topics among the key informants included perceptions regarding the design, feasibility, and scalability of the intervention.

### Data analysis

During data analysis, saturation was assessed iteratively by examining the extent to which new data contributed novel insights. When fewer than 5% of emerging codes reflected new information, we considered that the saturation point had been reached. All audio recordings from FGDs and KIIs were transcribed verbatim. Transcripts for AGYW were translated from the local language into English, and all of them were anonymized and stored on a password-protected drive before data analysis. We conducted thematic analysis using ATLAS.ti version 24 as per the socioecological model domains.

A preliminary codebook was collaboratively developed, refined, and then applied across all transcripts. Through constant comparison, similar codes were grouped into categories, which were subsequently mapped onto the five domains of the SEM. This ensured analytic coherence between emergent data patterns and the theoretical orientation of the study. Higher-level interpretation involved identifying relationships across the domains of the socioecological model, exploring convergent and divergent perspectives among AGYW to examine how structural, social, institutional, interpersonal, and individual factors would interact to shape intervention implementation. We maintained analytical memos and reflexive notes throughout to capture researchers’ insights, challenge assumptions, and enhance interpretive depth. Regarding rigor and trustworthiness, credibility was strengthened through triangulation of FGDs and KIIs, peer debriefing with the research team, maintenance of an audit trail documenting key methodological decisions, and ongoing reflexivity to minimize interpretive bias.

Representative quotes were selected to illustrate themes, preserve participants’ voices, and enhance contextual richness. Reporting the findings was guided by the COREQ (Consolidated Criteria for Reporting Qualitative Research) checklist [11] (S1 File).

### Ethics statement

Ethical approval was obtained from the Clarke International University Research Ethics Committee (Ref: CLARKE-2024–1113) and the Uganda National Council for Science and Technology (Ref: HS4697ES). Administrative clearance was granted by the Moroto District Health Office. Written informed consent was obtained from all adult participants. For participants unable to read or write, consent information was provided orally in the *Ngakarimojong* language, and verbal informed consent was obtained and documented by the research team and witnessed by a trained research assistant. For AGYW below 18 years, written parental or guardian consent was obtained in addition to written or verbal assent from the adolescent participant. To minimise the risk of coercion or undue influence, the consent process emphasised voluntariness, confidentiality, and the participant’s right to decline or withdraw at any time without consequences. Participants were given adequate time to ask questions and make informed decisions. KIIs and FGDs were conducted in private settings to minimize harm. Confidentiality was ensured through anonymization of transcripts, secure password-protected data storage, and restricted access to study files.

## Results

We conducted six FGDs involving a total of 51 AGYW and five KIIs with stakeholders at the district and health facility levels. Among the five key informants (Table 1), three were female, within the 40–44 and 45–49 age ranges. Most had held their current positions for at least nine years, approximately four had ten or more years of professional experience, and three possessed a master’s degree.

**Table 1:** Characteristics of key informants.

Key informants	Sex	Age (years)	Years in current position	Years of SRH experience	Years lived in region	Highest level of education
A	Male	45 - 49	9	18	30	Masters
B	Female	40 - 44	6	10	32	Masters
C	Female	45 - 49	9	5	20	Masters
D	Female	45 - 49	10	10	25	Bachelors
E	Male	40 - 44	12	20	26	Diploma

Of the 51 AGYW participants (Table 2), most 62.7% (32/51) were aged 18 years and above, single or never married (52.9%, 27/51), and had attained at least secondary education (39.2%, 20/51). Over half (52.9%, 27/51) were out-of-school, and the majority identified as Christians (78.4%, 40/51). In addition, 54.9% (28/51) had one to two previous births, and 84.3% (43/51) were employed or engaged in income-generating activities.

**Table 2:** Characteristics of the adolescent girls and young women.

Characteristic	Frequency (n = 51)	Percentage
**Age in years**		
<18	19	37.3
≥18	32	62.7
**Marital status**		
Married	18	35.3
Single	27	52.9
Divorced	6	11.8
**Schooling status**		
In-school	24	47.1
Out-of-School	27	52.9
**Highest level of education**		
None	6	11.8
Primary	18	35.3
Secondary	20	39.2
Tertiary	7	13.7
**Parity**		
None	19	37.3
1-2	28	54.9
≥3	4	7.8
**Employment status**		
Employed	8	15.7
Not employed	43	84.3
**Religion**		
Christian	40	78.4
Muslim	11	21.6

### Barriers and facilitators to implementation of the community-based, peer-led SRH education intervention

Table 3 presents the barriers and facilitators to implementing the community-based peer-led SRH education intervention across the domains of the SEM. At the individual and interpersonal levels, barriers such as stigma, limited autonomy, and social judgment emerged, while peer trust and supportive relationships served as important facilitators. At the institutional level, judgmental provider attitudes and confidentiality concerns were noted as barriers, contrasted with the availability of youth-friendly platforms as key facilitators. Community-level barriers included restrictive cultural norms and widespread myths about contraception, whereas community dialogues and local champions were identified as enabling factors. At the policy level, although no barriers were reported, existing supportive SRH policies and partnerships were viewed as important facilitators of the intervention’s implementation.

**Table 3:** Barriers and facilitators to implementation of the community-based, peer-led SRH education intervention.

Socio-ecological domain	Barriers	Facilitators
**Individual**	Internalised stigma and anticipated judgement (fear of being labelled, shame, self-censorship)	Confidence derived from peer-connected identity
**Interpersonal**	Parental, peer, and partner judgement and control Limited decision-making power over SRH	Peer trust and relational support Supportive family members and peer encouragement
**Institutional**	Judgmental attitudes among healthcare providers Concerns about breaches of confidentiality	School SRH clubs, and (Non-governmental organisation (NGO) and partner outreaches
**Community**	Myths and misconceptions Restrictive cultural norms	Community dialogues and youth forums Community champions
**Policy**		Supportive SRH policy and ongoing partnerships

### A. Individual-level barriers and facilitators

#### Stigma surrounding SRH.

At the individual level, stigma surrounding SRH did not operate merely as a personal attitude but as anticipated social judgment that shaped how AGYW managed visibility, reputation, and risk. Drawing on stigma process theory, anticipated labeling (“spoilt,” “bad girl,” “sexually active”) functioned as a pre-emptive regulatory mechanism: girls anticipated status loss and social exclusion and therefore self-censored or withdrew from SRH discussions and peer-led spaces before any direct negative encounter occurred. This dynamic was more pronounced among younger (15–17 years) and in-school participants, for whom school environments intensified visibility and peer surveillance.

One adolescent captured this reputational risk calculus:

*“(...) I feel ashamed to go for family planning because people will say I am already spoilt…” (Adolescent, 15–17 years, FGD 1)*

Rather than reflecting individual “shyness,” this demonstrates how community moral norms become internalized as shame, narrowing the range of “safe” behaviors and producing self-exclusion from intervention entry points (peer sessions and referral use). These individual responses were not isolated: participants’ fear of being “seen” links anticipated stigma to community-level norms and sanctions around premarital sex and contraception and to institutional settings where adolescents are exposed to adult scrutiny (schools and facilities).

#### Judgment from parents, elders, and peers.

Judgment from parents, elders, and peers is not just isolated criticism but part of a broader moral order. Girls internalize community norms that label adolescent SRH engagement as deviant and then police themselves to avoid that label. In school, one girl explained,

‘*if you are seen asking about family planning… other students start saying you are sexually active… you do not want to be labelled.’ (School-going girl, FGD 3).*

Such anticipated labeling (‘spoilt,’ ‘bad girl’) acts as a reputational risk. Faced with this social sanction, girls manage their identities by withdrawing from sessions and SRH discussions. This mechanism, community norms, interpersonal enforcement, and individual self-exclusion show how moral judgments become embodied as self-censorship.”This reputational concern was particularly salient among school-going girls, where peer scrutiny and institutional oversight intersect.

#### Peer trust.

Our data show that existing trust among peers emerged as a facilitator for intervention implementation. Adolescents reported that they often rely on peers for sensitive advice, suggesting that peer credibility and shared experience could enhance participation and sustain the intervention.

*“With my friends, I can ask anything without feeling ashamed.” (AGYW, 18–24, out of school).*

This facilitator was more strongly expressed among older (≥18 years) and out-of-school AGYW, who appeared to rely more on peer networks as primary sources of information and support.

### B. Interpersonal level barriers and facilitators

#### Limited decision-making power over contraceptive use and health-seeking.

Within the interpersonal domain, limited decision-making power over contraceptive use and health-seeking were identified as key barriers. Family members, especially parents and male partners, were perceived to exert considerable control, often restricting girls’ movements and participation in SRH programs. These constraints were more restrictive among younger adolescents and those still living under parental authority, while partner influence was more evident among older and ever-pregnant AGYW.

*“… if a man says no, you cannot get contraceptives …“(FGD, AGYW- <18).*

These findings illustrate how individual intentions are mediated and often overridden by relational power structures and demonstrate that barriers to SRH access are not simply about knowledge deficits but are embedded in negotiated and unequal social relations.

#### Supportive family and friends, and peer encouragement.

Supportive groups differed fundamentally from judgmental groups in both their social positioning and their influence on AGYW’s engagement with SRH interventions. Judgmental groups, including some parents, elders, and healthcare providers, functioned as moral gatekeepers who reinforced stigma, surveillance, and social sanctions around adolescent sexuality. Their responses were characterised by criticism, labelling, restriction of movement, and moralising attitudes that discouraged AGYW from participating in peer-led SRH activities or seeking services. In contrast, supportive groups, such as trusted peers, supportive family members, community champions, school SRH clubs, and youth-friendly providers, created socially affirming environments that reduced fear, shame, and isolation. These groups facilitated participation by normalising SRH discussions, encouraging collective engagement, and strengthening adolescents’ confidence to seek information and services. Analytically, this distinction suggests that implementation strategies should not treat communities as uniformly resistant or supportive. Rather, interventions should intentionally leverage supportive actors as entry points for trust-building and peer mobilisation, while simultaneously targeting judgmental actors through community sensitisation, provider training, confidentiality strengthening, and norm-change approaches aimed at reducing stigma and restrictive social control.

*“Sometimes we go as a group…, so that no one feels shy.” (AGYW, 18–24, in school).*

These dynamics point to the importance of collective support systems in enabling engagement, where shared experiences and mutual reinforcement create pathways for action that might otherwise remain inaccessible.

### C. Institutional-level barriers and facilitators

#### Judgmental attitudes among healthcare providers.

At the institutional level, judgmental attitudes among healthcare providers and concerns about breaches of confidentiality were reported as barriers that could limit girls’ willingness to use referral services provided through the peer program. Institutional barriers were not limited to service availability but were deeply embedded in provider attitudes and practices that reproduce societal norms. Health workers were described as moral gatekeepers, whose judgemental interactions reinforced stigma and deterred future care-seeking. These experiences were reported across all groups but were particularly discouraging for younger and first-time service seekers, who had limited prior interaction with the health system.

*“… preaching morals rather than providing services” (KI, district stakeholder-3).*

These encounters illustrate how institutional environments can legitimise and amplify community-level stigma, transforming health facilities into spaces of surveillance rather than care.

#### Concerns about breaches of confidentiality.

Concerns about breaches of confidentiality at health facilities emerged as a key institutional barrier to the implementation of the intervention. Participants reported that some health providers disclose adolescents’ health information to parents, undermining trust in the health system. This discourages AGYW from seeking services, even when they have been successfully mobilised through peer-led community activities. As one participant noted,

*“… when we go to the facility, we encounter health providers who cannot maintain confidentiality… they even tell our parents all our health issues, and that discourages us from coming back” (FGD, in-school AGYW).*

This highlights how institutional failures interact with interpersonal relationships, particularly family dynamics, to constrain access.

#### SRH clubs, and NGO, and partner outreaches.

Conversely, youth-friendly services, school SRH clubs, and NGO-supported outreach activities were cited as strong facilitators. These platforms were seen as safe, non-judgmental spaces for learning and for linking in- and out-of-school girls to SRH services.

These platforms were particularly important for in-school AGYW, while outreach services were noted to improve access for out-of-school and rural adolescents.

*“We usually go to the teenage center ……. so, we feel free to talk and ask questions” (AGYW, 18–24, in school).*

These spaces function as critical points of entry into care, where trust and accessibility intersect to translate awareness into actual service uptake.

### D. Community-level barriers and facilitators

#### Myths and misconceptions.

At the community level, myths and misconceptions, for instance, that contraceptive use causes infertility or encourages promiscuity, were recognized as substantial barriers to community acceptance. Many AGYW reported that contraception was considered acceptable only after a woman had demonstrated her fertility. Another commonly cited belief was that family planning “burns a woman’s eggs” and leads to infertility, hence preventing future childbearing. These beliefs were embedded in broader concerns about fertility, marriage, and social status, influencing both AGYW and gatekeepers such as partners and parents. Such misconceptions were especially influential among younger and never-pregnant AGYW, who had not yet interacted with formal SRH services.

*“… family planning burns eggs “ (AGYW, never given birth).*

These narratives reveal how shared beliefs shape perceived risks and priorities, reinforcing caution around contraceptive use and constraining decision-making beyond the individual level.

#### Restrictive cultural norms.

Additionally, deep-seated cultural norms discouraging open discussions about sexuality were also perceived to limit support for the intervention. For example, the culture of having many children has the potential to limit the intervention uptake. These norms illustrate how community expectations are internalised and enacted across multiple levels, shaping both individual behaviour and institutional responses. These norms were consistently reported across all strata, though their effects were more strongly enforced among younger girls, whose behaviour was more tightly regulated.

*“… having many children and any attempt to limit or space births is a threat to cultural values” (ID1, District stakeholder-3).*

*“… early childbearing is a marker of femininity and family prosperity” (KI, District stakeholder-2).*

These perspectives underscore how reproductive expectations are socially reinforced, creating a normative environment that constrains alternative choices and shapes what is considered acceptable behaviour.

#### Community dialogues and youth forums.

Community dialogues and youth forums were viewed as valuable facilitators to catalyze entry points for sensitization that would facilitate the implementation of the intervention. Community-level youth forums and dialogues provide safe, collective spaces for adolescents to share experiences, ask questions, and learn from peers and health workers. Participants described these forums as affirming, reducing feelings of isolation, and fostering solidarity among young people facing similar challenges. These platforms were particularly beneficial for out-of-school AGYW, who had fewer alternative spaces for SRH engagement.

*‘The dialogues help us to know we are not alone“(AGYW, 18–24, out-of-school).*

These youth forums thus function both as safe spaces for peer affirmation and as bridges between communities and formal health systems, reinforcing the effectiveness of peer-led approaches at scale.

#### Community champions.

The presence of community champions, including respected adults and peer leaders, was reported to enhance community trust, reduce backlash, and legitimize the peer-led approach. This was believed to facilitate the intervention implementation. The role of community champions further demonstrates how norm change is socially mediated. Endorsement by respected leaders conferred legitimacy on SRH interventions, reducing resistance and enabling broader acceptance. Community champions’ influence was noted across all groups, but was particularly important in shifting parental and community acceptance, thereby indirectly improving access for younger adolescents.

*“If a local council leader or elder says it is right for girls to seek family planning, the whole community listens. That is when change starts” (KI, stakeholder-1).*

Indeed, AGYW recognized the value of supportive champions and noted that their endorsement would reduce shame and make it easier for them to seek SRH services. Many AGYW indicated that community-level approval from respected figures was not symbolic alone but translated into real shifts in their willingness to engage with SRH programs.

### E. Policy-level barriers and facilitators

At the policy level, we did not identify potential barriers to the intervention. However, supportive SRH policy and partnerships emerged as a facilitator.

#### Supportive SRH policy and ongoing partnerships.

Supportive national adolescent SRH policies and ongoing partnerships with NGOs and community actors created an enabling environment for implementation of the peer-led intervention. This suggests that policy operates as a necessary but not sufficient condition, requiring effective translation into practice through health systems, community structures, and social relations. Participants across all strata emphasised that policy benefits were unevenly experienced, depending on how effectively they were implemented at the facility and community levels.

*“Government emphasis [policies and guidelines] on youth-friendly services and partnerships with NGOs is what makes it possible to reach many young girls with SRH information and care” (KI,2).*

These insights highlight that the effectiveness of policy depends on how well it is operationalised and integrated across systems, with implementation realities ultimately determining its reach and impact*.*

## Discussion

This study explored the barriers and facilitators to the implementation of a community-based, peer-led SRH intervention among AGYW in northeastern Uganda. Rather than treating these factors as discrete “levels,” we interpret them as interacting processes that shape whether AGYW can safely engage with SRH information and change their sexual behaviour. The Socio-ecological model emphasizes that sexual behavior and SRH services use are produced through multiple, mutually influencing contexts (intrapersonal, interpersonal, organizational/institutional, community, and policy), making implementation success dependent on alignment across these domains [12,13]. At the individual level, stigma surrounding SRH discussions emerged as a key barrier to the implementation of the intervention. Our findings indicate that stigma, judgement, and trust operate not merely as individual attitudes, but as relational and socially produced dynamics that regulate adolescent sexuality. AGYW feared that attending peer sessions or seeking contraception would result in being labelled “spoilt” or morally deviant, echoing evidence from across sub-Saharan Africa showing stigma as a deterrent to health-seeking behavior [12]. Similarly, evidence from Ghana demonstrates that high levels of SRH-related stigma reduce service uptake [13]. Interpreted analytically, this “fear of attendance” reflects not simply personal hesitation, but an anticipation of social sanction in contexts where adolescent sexuality is closely monitored and morally regulated. Such dynamics may limit participation, reduce receptivity to information, and undermine consistent engagement with the intervention.

These stigma processes are reinforced across levels. At the interpersonal level, limited decision-making power over contraceptive use and health-seeking reflects broader age- and gender-based power relations. Our findings are consistent with evidence from Kenya showing that parental disapproval and lack of family support restrict adolescents’ access to contraception and SRH information [14]. In our study, some parents viewed SRH discussions as inappropriate for unmarried girls, a belief similarly documented in Western Kenya, where elders discouraged adolescents from accessing contraception on the basis that it promotes promiscuity [15]. Such gatekeeping extends beyond “lack of support” to represent a relational mechanism through which adults regulate adolescent sexuality, constraining enrolment, disrupting continuity, and limiting intervention reach.

At the same time, peer support emerged as a key relational facilitator of implementation. AGYW described how encouragement from peers increased their willingness to engage in the intervention. Evidence indicates that peer-led approaches improve SRH knowledge, attitudes, and confidence to seek services [16], while a community-based trial in Zambia demonstrated increased SRH service uptake through peer networks [17]. These findings suggest that peer groups can function as trusted social spaces that mitigate fear and normalize engagement with SRH information. However, peer trust alone may be insufficient if broader social and institutional constraints remain unaddressed.

At the institutional level, healthcare provider attitudes and confidentiality concerns emerged as critical barriers that may undermine implementation. AGYW reported experiences of being scolded, judged, or morally lectured by healthcare providers, discouraging them from seeking SRH services. Existing literature reinforces that judgmental communication and discriminatory behaviour by health workers deter adolescents from utilizing services [18], while disrespectful treatment and inadequate privacy reduce confidence in youth-friendly services [19]. Broader evidence across sub-Saharan Africa highlights persistent gaps in adolescent SRH service delivery, including fragmented services, inadequate provider training, and compromised confidentiality [20]. Adolescents consistently prefer services that ensure privacy and respectful, non-judgmental care and disengage when these elements are absent [21].

Confidentiality concerns further illustrate how trust operates as a relational process shaping service uptake. AGYW feared that healthcare providers might disclose sensitive information to parents or community members, particularly in close-knit rural settings where information spreads quickly. Similar findings from other low-resource contexts show that perceived risks of privacy breaches significantly deter adolescents from accessing SRH services [22].

In this context, such concerns undermine the intervention’s referral pathways, as AGYW may avoid follow-up services despite participating in peer sessions. Strengthening confidentiality safeguards and rebuilding trust in provider discretion will therefore be essential for effective implementation.

At the community level, myths and misconceptions about contraception, particularly fears that modern methods cause infertility, emerged as significant barriers. These beliefs reflect socially embedded narratives that shape perceptions of contraceptive safety and acceptability. Similar findings from South Africa show that fears of side effects and infertility undermine contraceptive uptake among adolescents [23]. In our study, contraception was often viewed as acceptable only for women who had “proven fertility,” reinforcing cultural expectations around early childbearing. Such norms not only perpetuate stigma but also generate community resistance, limiting support for peer-led programming and discouraging participation. However, community engagement also emerged as a facilitator. AGYW noted that the involvement of elders, community dialogues, peer networks, and endorsement by local leaders increased trust and legitimized the intervention within the community. This finding aligns with a growing body of implementation and adolescent SRH scholarship in sub-Saharan Africa demonstrating that community-based participatory approaches, social norm interventions, and engagement of influential community actors can improve the acceptance of adolescent contraceptive use and strengthen the uptake of SRH services [23,24]. Studies conducted in South Africa, Kenya, Zambia, and Nigeria have shown that when trusted leaders, peer networks, and community structures actively support SRH initiatives, adolescents are more likely to perceive service access as socially acceptable and safe. Within the study setting, community engagement functioned not only as a sensitization strategy but also as a mechanism for reducing stigma, challenging misconceptions, and increasing collective ownership of the intervention..

At the policy level, supportive national adolescent SRH frameworks provide an enabling environment for implementation. This aligns with evidence from Uganda and comparable contexts such as Nigeria, where national policies guide adolescent health programming [24]. However, the presence of policy support does not automatically translate into practice, suggesting the need to bridge gaps between policy commitments and lived service experiences.

Overall, our findings demonstrate that the implementation of a community-based, peer-led SRH intervention is shaped by the interaction of relational dynamics across multiple levels of the socio-ecological model. This aligns with an Africentric reproductive justice framework [25], which recognises that reproductive health outcomes are embedded within cultural, social, and historical contexts specific to African settings. Within this perspective, trust, stigma, power relations, and social norms operate across individual, interpersonal, institutional, and community domains, collectively influencing whether adolescent girls and young women (AGYW) can safely access and engage with SRH information and services. Programmatically, this implies that interventions must go beyond information delivery to address the relational conditions that influence participation and follow-through. This includes strengthening confidentiality safeguards, improving provider–adolescent interactions, engaging communities to shift norms, and addressing gendered power dynamics that constrain adolescent decision-making.

### Strengths and limitations

This qualitative study has several strengths. Exploring perspectives across multiple levels provides a rich, nuanced understanding of the complex environment influencing intervention implementation. The Socio-ecological model allowed for a systematic analysis of how different domains interact to affect AGYW’s SRH behaviors. Additionally, conducting the study in a hard-to-reach, post-conflict region contributes new insights that may be transferable to similar settings with high SRH needs and sociocultural sensitivities. The use of peers in data collection was an additional strength of this study. Peer researchers often elicit more open and honest accounts from AGYW because they reduce power imbalances, enhance rapport, and create a perceived safe space for discussing sensitive SRH topics.

This study has certain limitations to consider. Data were obtained from a single sub-region, Karamoja, whose distinct cultural and social context may limit wider applicability. Because the research preceded full intervention roll-out, participants described anticipated rather than actual implementation experiences, which might not reflect realities during delivery. Some social desirability bias is likely, particularly around sensitive SRH behaviours. The absence of perspectives from adolescent boys also narrows the interpretation of community norms. Trained research assistants served as witnesses; however, we acknowledge that they may not be entirely independent. Finally, although peer-led SRH approaches are supported in other settings, this model has not been previously adapted to this context, and its acceptability and feasibility in practice remain to be established.

## Conclusion and recommendations

Implementation of the community-based peer-led SRH education intervention among AGYW will be influenced by factors across individual, interpersonal, institutional, community, and policy levels. Future implementation should consider reducing stigma, judgmental healthcare provider attitudes, and cultural barriers while promoting peer trust, supportive relationships, and youth-friendly services to enhance implementation. Additionally, strengthening partnerships and coordination within the existing supportive policy environment will be essential to sustain implementation and improve SRH outcomes among AGYW in the setting. Future studies will leverage these facilitators and address the potential barriers to enhance implementation.

## Supporting information

S1 File Contains completed 32-item COREQ checklist for reporting qualitative research methods and findings, covering domains of research team characteristics, study design, data collection procedures, analysis, and reporting. Corresponding manuscript page numbers are provided for each item.
(DOCX)

S1 Data Contains raw qualitative data from 5 Key Informant that are district stakeholders involved in SRH programming and Focus Group Discussions (FGDs) with adolescent girls and young women (AGYW). Anonymised verbatim excerpts from six FGDs (n = 51 participants), stratified by age (15–17 years; 18–24 years), schooling status (in-school; out-of-school), and parity (ever given birth; never given birth). Data are organised thematically and reflect participants’ perspectives on barriers and facilitators to implementing a peer-led SRH intervention.
(PDF)

## References

[1] Uganda K. Uganda Bureau of Statistics. Kampala, Uganda and Calverton; 2016.

[2] Ayoo S, Opio R, Kakisa OT. Karamoja situational analysis. Northern Uganda Women’s Empowerment Programme (NUWEP). Kampala: CARE International in Uganda; 2013.

[3] Scott-Villiers P. Mechanisms of securitisation and counter-securitisation on the Uganda-Kenya border. Confl Secur Dev. 2025;25(2):133–55. doi: 10.1080/14678802.2025.2459410

[4] Chandra-Mouli V, Lane C, Wong S. What does not work in adolescent sexual and reproductive health: a review of evidence on interventions commonly accepted as best practices. Glob Health Sci Pract. 2015;3(3):333–40. doi: 10.9745/GHSP-D-15-00126 26374795 PMC4570008

[5] Krishnaratne S, et al. Acceptability of family planning in a changing context in Uganda: a realist evaluation at two time points. BMJ Open. 2022;12(4):e054277. 10.1136/bmjopen-2021-054277 PMC8995957 35396286

[6] Unesco. Global education monitoring report 2020: inclusion and education-all means all. UN; 2020. 10.1007/s11125-020-09505-x PMC7498984 32963414

[7] Achen S, Atekyereza P, Rwabukwali CB. The role of culture in influencing sexual and reproductive health of pastoral adolescent girls in Karamoja sub-region in Uganda. Pastoralism. 2021;11(1):25. doi: 10.1186/s13570-020-00188-9

[8] Creswell JW, Miller DL. Determining validity in qualitative inquiry. Theory Pract. 2000;39(3):124–30. doi: 10.1207/s15430421tip3903_2

[9] Carlsen B, Glenton C. What about N? A methodological study of sample-size reporting in focus group studies. BMC Med Res Methodol. 2011;11:26. doi: 10.1186/1471-2288-11-26 21396104 PMC3061958

[10] Bronfenbrenner U. The ecology of human development: experiments by nature and design. Harvard University Press; 1979.

[11] Tong A, Sainsbury P, Craig J. Consolidated criteria for reporting qualitative research (COREQ): a 32-item checklist for interviews and focus groups. Int J Qual Health Care. 2007;19(6):349–57. doi: 10.1093/intqhc/mzm042 17872937

[12] McLeroy KR, Bibeau D, Steckler A, Glanz K. An ecological perspective on health promotion programs. Health Educ Q. 1988;15(4):351–77. doi: 10.1177/109019818801500401 3068205

[13] Golden SD, Earp JAL. Social ecological approaches to individuals and their contexts: twenty years of health education & behavior health promotion interventions. Health Educ Behav. 2012;39(3):364–72. doi: 10.1177/1090198111418634 22267868

[14] Harrington EK, et al. “Spoiled” girls: understanding social influences on adolescent contraceptive decision-making in Kenya. PloS One. 2021;16(8):e0255954. 10.1371/journal.pone.0255954 PMC8360567 34383836

[15] Mwaisaka J, Wado YD, Ouedraogo R, Oduor C, Habib H, Njagi J, et al. “Those are things for married people” exploring parents’/adults’ and adolescents’ perspectives on contraceptives in Narok and Homa Bay Counties, Kenya. Reprod Health. 2021;18(1):48. doi: 10.1186/s12978-021-01107-w 33622358 PMC7903790

[16] Mason-Jones AJ, Freeman M, Lorenc T, Rawal T, Bassi S, Arora M. Can peer-based interventions improve adolescent sexual and reproductive health outcomes? An overview of reviews. J Adolesc Health. 2023;73(6):975–82. doi: 10.1016/j.jadohealth.2023.05.035 37452795 PMC7615313

[17] Phiri MM. The impact of an innovative community-based peer-led intervention on uptake and coverage of sexual and reproductive health services among adolescents and young people 15–24 years old: results from the Yathu Yathu cluster randomised trial. BMC Public Health. 2024;24(1):1424. 38807091 10.1186/s12889-024-18894-z PMC11134624

[18] Onukwugha FI, Hayter M, Magadi MA. Views of service providers and adolescents on use of sexual and reproductive health services by adolescents: a systematic review. Afr J Reprod Health. 2019;23(2):134–47. doi: 10.29063/ajrh2019/v23i2.13 31433601

[19] Ninsiima LR, Chiumia IK, Ndejjo R. Factors influencing access to and utilisation of youth-friendly sexual and reproductive health services in sub-Saharan Africa: a systematic review. Reprod Health. 2021;18(1):135. doi: 10.1186/s12978-021-01183-y 34176511 PMC8237506

[20] Sanyang Y, Sanyang S, Ladur AN, Cham M, Desmond N, Mgawadere F. Are facility service delivery models meeting the sexual and reproductive health needs of adolescents in Sub-Saharan Africa? A qualitative evidence synthesis. BMC Health Serv Res. 2025;25(1):193. doi: 10.1186/s12913-025-12344-1 39893420 PMC11786442

[21] Uka VK, White H, Smith DM. The sexual and reproductive health needs and preferences of youths in sub-Saharan Africa: a meta-synthesis. PLoS One. 2024;19(12):e0300829. doi: 10.1371/journal.pone.0300829 39739925 PMC11687907

[22] Halper E, Erhardt-Ohren B, Cobb M, Hidalgo-Mora O, Ospina-Henao S, O’Bannon A, et al. Socio-ecological influences on access to abortion care in Costa Rica: a qualitative analysis of key perspectives from clinical and policy stakeholders. Sex Reprod Health Matters. 2024;32(1):2374137. doi: 10.1080/26410397.2024.2374137 39105442 PMC11305048

[23] Jonas K, et al. Rumours, myths, and misperceptions as barriers to contraceptive use among adolescent girls and young women in South Africa. Front Reprod Health. 2022;4:960089. 36406890 10.3389/frph.2022.960089 PMC9673823

[24] Arije O, Hlungwani T, Madan J. Key informants’ perspectives on policy- and service-level challenges and opportunities for delivering adolescent and youth-friendly health services in public health facilities in a Nigerian setting. BMC Health Serv Res. 2022;22(1):1493. doi: 10.1186/s12913-022-08860-z 36476291 PMC9727905

[25] Mulumba M, Oga J, Muhumuza N. Beyond reproductive rights: implementing the Africentric reproductive justice framework in sexual and reproductive health and rights litigations in Africa. Sex Reprod Health Matters. 2025;33(1):2570579. doi: 10.1080/26410397.2025.2570579 41070611 PMC12692456

