# Supplementary material for: Barriers and facilitators to community-based, peer-led sexual and reproductive health intervention for adolescent girls and young women in northeastern Uganda: A qualitative study
Source: PLOS Glob Public Health. 2026 Jun 11;6(6):e0005828. doi: 10.1371/journal.pgph.0005828 (PMC13257979; doi:10.1371/journal.pgph.0005828)
Supplement: S1 Data — Anonymised verbatim excerpts from six FGDs (n = 51 participants), stratified by age (15–17 years; 18–24 years), schooling status (in-school; out-of-school), and parity (ever given birth; never given birth). Data are organised thematically and reflect participants’ perspectives on barriers and facilitators to implementing a peer-led SRH intervention. (PDF) [file pgph.0005828.s002.pdf]

To explore the contextual factors that might influence the implementation of CPBL SRH Education among AGYW in Moroto District Findings from the Focus group discussions

We conducted six focus group discussions (FGDs) involving a total of 51 AGYW and five key informant interviews with stakeholders at the district and health facility levels.

Among the five key informants (Table 1), three were female, the oldest was 47 years old, and the youngest was 42 years old. Most had held their current positions for at least nine years, approximately four had ten or more years of professional experience, and three possessed a master's degree.

Table 1: Characteristics of key informants

Key informants	Sex	Age (years)	Years in current position	Years of SRH experience	Years lived in region	Highest level of education
A	Male	47	9	18	30	Masters
B	Female	43	6	10	32	Masters
C	Female	46	9	5	20	Masters
D	Female	45	10	10	25	Bachelors
E	Male	42	12	20	26	Diploma

Characteristics of the respondents

The majority of AGYW were 17 years and below, 41.2% (21/51), with most being single, 62.7% (32/51). Slightly more than half were out of school, 52.9% (27/51), and the largest group had completed secondary education, 39.2% (20/51). Regarding parity, the highest proportion had no 1-2 children, 54.9% (28/51). The vast majority were not employed, 84.3% (43/51), and most identified as Christian, 78.4% (40/51) as indicated in table 2.

Table 2: Characteristics of the Adolescent Girls and Young Women

Characteristic	Frequency N= 51	Percentage
**Age**		
17 years and below	21	41.2
18- 21 years	16	31.4
22-24 years	14	27.4
**Marital status**		
Married	13	25.5
Single	32	62.7
Divorced	6	11.8
**Schooling status**		
In school	24	47.1
Out of School	27	52.9
**Highest level of education**		
None	6	11.8
Primary	18	35.3
Secondary	20	39.2
Tertiary	7	13.7
**Parity**		
None	19	37.3
1-2	28	54.9
3-4	4	7.8
**Employment status**		
Employed	8	15.7
Not employed	43	84.3
**Religion**		
Christian	40	78.4
Muslim	11	21.6

Source: Primary data from AGYW

Category 1: Perceived facilitators and barriers

Participants welcomed and valued with importance the idea of a community-based peer-lead sexual and reproductive health education in Moroto. They appreciated the innovation of peer services which were none existent to cater for the sexual and reproductive health needs of the adolescent and young women population in the area. Participants valued peer support because they thought it will be the easy and effective way of getting many youths into seeking and utilising sexual and reproductive health services. Peer service delivery models are looked at as ways that empower adolescent girls and young women to take control over concerns of their own health and wellbeing.

The trust that AGYWs have in their peers was a strong point to facilitate the delivery of SRH education through a community-based peer-lead delivery model.

"Most times I get advice from friends when I have big issues disturbing my mind because I trust my friend" **(AGYW, age 18-24, in school)**.

Availability of Sexual and Reproductive health services was perceived to greatly facilitate the community-based peer lead SRH education services. Participants argued that it is easy when you educate adolescents and direct them to where they can receive services and the services are readily available for them. SRH services are said to be available in Moroto Regional Referral Hospital, other health centres like Nakapelimen and in schools. Importantly, most health facilities have youth conners or teenage centres where AGYW receive friendly services including SRH services tailored to their needs.

> *"We obtain SRH services from the teenage center at Moroto RRH or from Nakapelimen health center"* ***(AGYW, age 18-24, in school).***

Participants observed that for a community-based peer-lead SRH education intervention to thrive, it will leverage on the AGYW's wide awareness of the existence of SRH services in the district. This is facilitated by easy access to information about SRH services. Information about SRH is mainly got from school, health facilities, parents, boyfriends, peers or friends. Additionally, participants perceived themselves to be at high risk of unwanted pregnancies due to the nature of a community that is characterised with too much alcohol abuse, theft, and poverty that makes every young girl to opt for marriage at an early age. Moreover, AGYWs in this region felt empowered to make a decision or change a decision made by anyone else on their behalf about sexual and reproductive health.

> *"Most young people are aware of availability of services especially those in school because they have different ways to get information. For example, at school we have a senior woman teacher who gives us information"* ***(Girl, age 15-17, in school).***

Whereas participants largely described the possible facilitators to a community-based peer-lead SRH education, some participants shared thoughts about the possible barriers to this kind of model. Basically, their concerns rotated around stigma and disclosure of one's health condition or status by the health care providers or peers. Although there is trust in the peers to deliver SRH services, some AGYWs did not support

the use of peer lead delivery SRH education

because they cannot trust peers with secrets about their reproductive health since they are not trained to a level of health care workers whose profession and training guarantees safety of patient's information. The other concern was limited access to the correct and right information about SRH. Participants explained that whereas AGYWs have a variety of sources to information about SRH, not all sources are authentic. They get a lot of misconceptions and myths that will hamper the community-based peer-lead SRH service delivery.

> *"There is a lot of awareness among AGYWs on SRH. The problem is getting the courage to go to hospital when you have a problem which is common for many of us because we fear people pointing at us or showing us bad attitude and telling the public about our problems"* ***(AGWY, given birth).***

> *"in Moroto District, most youth are aware of the value of SRH services especially those who are educated. The only problem is that in our community, many youths cannot access correct information to be able to make a correct decision. Sometimes girls say this is bad based on a myth"* ***(Girl, age 18-24, in school).***

Another barrier foreseen to affect the effectiveness of a community-based peer-lead SRH education, is lack of time to attend such sessions by the AGYW. Given their assigned gender roles, as girls they spend most of the time doing domestic chores including; cooking, taking care of siblings, fetching water among others. This leaves them with limited time if any to attend hospitals or going to places where sessions may have been organised.

> *"Sometimes our parents give us a lot of work and even punish you when you refuse. At times when you say you want to go to hospital they simply think, that you want to go and visit a boyfriend yet at times you don't want them to know the type of disease that you suffering from"* ***(Girl, age 15-17, in school).***

Category 2: AGWYs SRH decision making

Power dynamics in the family under which AGYWs subscribe determines the

AGYW's decision towards SRH in Moroto. A parent, elder sibling, partner or boyfriend to the AGYW possess more power than the adolescent girl herself and influences the decision an adolescent girl or young woman will take towards SRH. For example, while making decisions to use or not, what

type to use of family planning, whether to use or not to use contraceptives, taking an HIV test is highly predisposed by parents and partners to the AGYW. Girls believe that they cannot refuse what their parents ask or even force them to do because they are still staying with me so they must have authority over what they do. Others, for fear of domestic violence being inflicted to them by their partners, they take the submissive side for better and continuity of their relationships.

> *"In many cases our parents influence our decisions in many ways and sometimes they force us to do certain things like getting married when you are still very young in order to bring wealth to the family"* ***(Girl never given birth).***

> *"Sometimes my decisions are influenced by friends or my boyfriend but this is the best choice because anything can happen between me and him if I did something without his knowledge" (**Girl ever given birth**).*

Category 3: Cultural perspectives

Cultural norms and values are big determinant for acceptability of SRH education lead by peers in Karamojja. For a girl marrying while you are still young is prestigious because it means the husband will put a lot of value to you. In addition, getting married to an elder man is highly treasured because it means such a girl is married to a rich man who will bring a big number of cows as dowry. Further still, for a girl getting married while is still a virgin, commands a lot of respect to her parents from the husband and his family. Some parents with an aim of obtaining such respect, they force their children into marriage while they are still young.

> *"When you marry an older man, he can take good care of you and can bring wealth to the family by giving them more animals"* ***(Girl never given birth).***

> *"The community here believes that when their daughters get married off as early as possible it brings respect to the parents especially if she is a virgin and that means more wealth into girl's family"* ***(Girl, age 15-17, in school).***

Family planning services including contraceptives are believed to be only for woman who have ever given birth because community members think that

contraceptives will make a girl barren or completely fail to have children in the future. It is stigmatizing for a girl in school and one who has never given birth to go to the health facility to seek family planning services or

contraceptives. Some participants viewed health workers as their parents since they are in the same age range and elders have equal respect like the biological parents so they are shy opening up to them about contraceptives because they will know they engage into sexual activities that is not expected when a girl is not married.

> *"People in this community think young people should not use family planning. It should be used by people who have had the children they want because it causes infertility"* ***(Girl, ever given birth****).*

Abortion is a taboo in Moroto District. Society discriminates any girl suspected to have aborted. It is shaming for any woman to abort regardless of the circumstances under which one conceived. It will be hard for a girl who has ever aborted to find a man to marry her. It is believed that abortion brings a curse into a family of such a girl committing abortion. This curse will claim lives of people moving forward.

> *"Most people hear treat women who have aborted with shame and they are discriminated because they have brought a curse to the family and that family members will start dying because of that curse"* ***(Girl, ever given birth).***

Men are the final decision makers in any marriage. In Moroto District, every decision made in a home must be made or supported by the husband. Most men do not want family planning because they want their wives to give birth to many children since children are a symbol of wealth. Specifically, girl children are seen as assets that will bring wealth to the parents in form of bride price during the process of marriage. Women will therefore fear to take contraceptives or family planning without permission from the husband hence sabotaging the effectiveness of SRH services.

> *"The only problem is that in our community of Moroto District, a man's decision in the home is final for the young women who are married so if a man says no, you cannot get contraceptives or go for family planning*" **(Girl, ever given birth).** *"The man here decides for his wife to go for family planning. The problem most men do not like some of these things"* ***(Girl, 18-24, in school)***.

Category 4: Recommendations

Participants made two major recommendations if a community-based peer-lead SRH education is to smoothly take place in Moroto. First, community sensitization aimed at changing the mind set of people in Karamoja about the benefits of SRH services. In some homes girls seeking SRH services are seen as wasted. It is believed that sensitization will help to deal with and degenerate some cultural beliefs attached to SRH services for AGYW in the region. Furthermore, sensitization will improve access to correct information about SRH to escape having girls driven by myths around SRH.

> *"You need to increase community sensitization on health problems and where young people can go for services. Help communities know that its not bad for a girl to receive such services" (**Girl, age 15-17, in school).***

The second is associated with stigma reduction training for the peers selected to lead the community-based SRH education. With is kind of training, peers will be able to avoid disclosure of fellow AGYW information if they approached them. This training too will help the service providers to use non-judgemental language and attitude that scare girls from going to facilities to access SRH services.

> *"Train health workers and those people in hospital, they should not scare us with the language of blaming us and showing us attitude that they do not want to work on us" **(Girl, ever given birth).***

There were also suggestions to have women handling SRH service delivery to the AGYW instead of men. Girls believe they can freely open up to a fellow woman than to a man. This is underpinned by the advice for hospitals or other health centres to emulate what is done in schools where girls' Reproductive health issues are handled by senior women not men.

> *"Female issues should be handled by women because it promotes opening up. I think it is the same reason why schools have senior one teacher" (**Girl, ever given birth).***

4.0 Findings from the Key informant Interviews with District Health Officer, Assistant District Health Officer, Community Development Officer and in chargers of healthcare facilities

Category 1: Barriers to Access SRH information

Many adolescent girls and young women (AGYW), especially in rural areas, primarily rely on friends for information on sexual and reproductive health (SRH), with limited access through conventional sources such as healthcare facilities or parents. Low literacy levels further hinder the effective flow of information. Media coverage, including radio and other outlets, has limited reach in rural regions, making it challenging for AGYW to access SRH information, even though initiatives like Resident District Commissioner airtime programs. In these communities, cultural myths and strong social beliefs act as barriers, obstructing AGYW from seeking or embracing SRH services. Many avoid these services due to misconceptions or negative perceptions they have heard or believe in. Additionally, AGYW in rural areas are less aware of the available SRH services and face infrastructural and social constraints that further limit access.

"The community in Moroto is highly conservative with low literacy levels, limiting AGYW's willingness to take on information. Most young women mainly get SRH information from friends, as parents in this enclosed system cannot discuss such topics with their children" ***(1DI-3).***

"Although SRH information is shared on the radio through Resident District Commissioner airtime, the radio coverage in the region is very low, making it difficult for many AGYW to access this information" ***(IDI-2).***

"Young people are aware that SRH services are available in hospitals, but the main challenge is the fear of being discovered with conditions like STDs, which discourages them from seeking care." ***(ID1-5).***

"Most youth in town are aware of SRH services, but their use is limited by attitudes, fear, lack of comprehensive knowledge and lack of drugs in the community health facilities ***(IDI -1).***

Category 2: Facilitators to Access to SRH information

In urban areas, adolescent girls and young women (AGYW) are generally more aware of available sexual and reproductive health (SRH) services and know where to access them, including hospitals, schools, and media outlets. Health workers confirm that SRH services such as counseling, family planning, and health education are accessible at certain health centers, with more advanced services provided at facilities like the "Teenage Center" in Moroto Regional Referral Hospital. Additionally, AGYW who can afford private services often seek SRH care at private clinics in urban municipalities. When provided with clear and accurate information, many AGYW are receptive to using SRH services, particularly in areas where access is more readily available.

> *"Here in Moroto, most AGYW get information on SRH when they come to the hospital, through radio and TV. Some of them get information on SRH through their teachers at school or from reading the Straight Talk newspaper"* ***(IDI-2).***

> *"Most youth embrace SRH services by using them when they need them. As a counsellor and nurse at the health center, every day when I am on duty, I attend to young people with different SRH needs"* ***(IDI-4).***

Category 3: Risky sexual behaviour among AGYW

Respondents observed that AGYW, except those from strong family backgrounds, engage in risky behaviors such as excessive drinking and unsafe sexual practices, with alcohol consumption impairing their judgment and influencing their ability to access SRH services when needed. They emphasized that peer-led, community-based education could be highly effective in addressing SRH issues in Moroto, given the strong community cohesion, especially if peer educators are trusted and well-known. Respondents stressed the importance of focusing SRH services on AGYW due to their high-risk lifestyles, which are marked by limited awareness of the consequences of their behaviors. Respondents noted that AGYW often share problems within closed circles and are more likely to accept incorrect information from friends over accurate information from health workers. Respondents strongly support the idea of community education as an appropriate strategy to improve SRH service utilization among AGYW in the district and also to reduce

risky sexual behaviour.

“Youth start drinking early at a young age and get involved in early sexual relationships with mature men." ***(IDI-4).***

*"Most young women marry older men, and the decision to use SRH services such as family planning is in the hands of their husbands. As a woman, you have nothing to say."(****1D1-3).***

*“There is a lot of drinking and bad sexual behavior. Young people start drinking when they are still children, and the society sees this as normal and a way of socialization."(****IDI-5).***

Category 4: Decision making power among AGYW

Respondents noted that AGYW in Moroto District face significant limitations in making their own decisions regarding sexual and reproductive health (SRH) due to economic hardships and reliance on parents, husbands, or multiple boyfriends for survival. This dependence compromises their autonomy, with decisions regarding family planning and marriage often made by men, parents, or clan leaders without consulting the AGYW. Marriage is commonly seen as an economic opportunity to gain wealth in the form of livestock, further diminishing AGYW's ability to make informed choices about their SRH. Peer influence, limited knowledge, and cultural norms also hinder their decision-making, particularly in rural areas where family and community dynamics dominate. The respondents acknowledge these challenges and suggests that, while community-based peer-led SRH models may be impacted by AGYW's low decision-making power, involving community leaders such as Local Council 1 members, elders, and trusted "champions" in ongoing community sensitization could promote greater acceptance and access to SRH services.

“A lot of teenage marriages are influenced by their parents, AGYWs have limited ability to make personal decisions even on their health” ***(1DI-3).***

“Some women have attempted to secretly use family planning without their husbands’ knowledge and this sometimes has led to instances of gender-based violence” ***(IDI-4).***

“The decision-making power is likely to affect SRH because many

young people may not be able to decide for themselves yet wrong information is very

popular" ***(IDI-1).***

"If this program is to succeed, we will need to work more with the health care system and community champions who are always good in delivering messages and are trusted by the community" ***(IDI-2).***

Cultural Barriers and Myths Surrounding SRH Services

Respondents noted that in the community, marriage is viewed as a way for families to gain wealth through livestock, leading to early marriages for young girls. Family planning is surrounded by negative myths, while abortion is strongly condemned. Despite this, some women still seek family planning services. Addressing these misconceptions could improve the acceptability of community-based peer-led SRH education. The respondents emphasized that marriage is seen as a way to enrich families and that cultural values conflict with family planning. They suggested that using influencers and satisfied users could promote the proposed intervention and improve SRH service acceptance.

"In this community, using family planning is like a head on collision with cultural values, some people think when you use family planning you may not have children anymore" ***(IDI-4).***

"Here people value large families and relate this to wealth. Socially abortion is perceived negatively and it is believed that family members will contiously die in that family where the abortion had been committed" ***(ID1-5).***

"Here in our community people look at marriage as a way of getting rich by giving away their daughters to a person who gives them more animals" ***(IDI-1).***

Integration of community-based peer-led SRH education into existing health programmes

Respondents noted that SRH services are available free of charge at the main Regional Referral Hospital and all health centers. Then other institutions like Marie stopes and Straight Talk Foundation. They believe that community-based, peer-led SRH education would likely be accepted, especially if the peer educators are well-known in the community. Integrating community-based peer-led SRH education into the existing health programmes is a positive step, as it could improve referrals from villages to hospitals.

Also eliminate barriers to SRH services at

the district level such as the long distances to health facilities and the limited supply of drugs from the national supply chain system.

> *"Most health facilities in the region offer SRH services but sometimes young people fear to come to hospital when they have problems. Other institutions that offer SRH services are Striaght talk foundation, Brac Uganda, save the children, Doctors with Africa, CUAAM, UNFPA and Marie stopes"* ***(IDI-1).***

> *"It is nice to hear about this program. I think this is a good idea and when we add it to the existing health programmes, we can get people referred for treatment from the community"* ***(IDI-3).***

> *"The community-based peer-led SRH education program will require to adopt the champions approach and integrate them into a team of service providers that are already existing in the formal health care system. This peer led approach will be more acceptable than other models"* ***(IDI-5).***

> *"Integrating gender sensitivity into the community-based peer-led SRH education model, by involving female and younger health workers, can help address barriers such as conservative cultural practices, religious influences, and long distances to health facilities"* ***(IDI-2).***
